# Supplementary material for: Bioinformatics Tools for NGS-Based Identification of Single Nucleotide Variants and Large-Scale Rearrangements in Mitochondrial DNA
Source: BioTech (Basel). 2025 Feb 12;14(1):9. doi: 10.3390/biotech14010009 (PMC11843820; doi:10.3390/biotech14010009)
Supplement: Supplementary file 1 [file biotech-14-00009-s001.zip › biotech-3369914-supplementary.pdf]

BioTech

MDPI

Article

Bioinformatics Tools for NGS-based Identification of Single Nucleotide Variants and Large-Scale Rearrangements in mitochondrial DNA

Marco Barresi [1], Giulia Dal Santo[1], Rossella Izzo[1,2], Andrea Zauli[1], Eleonora Lamantea[1], Leonardo Caporali[3], Daniele Ghezzi[1,2,*], Andrea Legati[1]

[1] Unit of Medical Genetics and Neurogenetics, Fondazione IRCCS Istituto Neurologico Carlo Besta, Milan, Italy

[2] Department of Pathophysiology and Transplantation (DEPT), University of Milan, Milan, Italy

[3] Laboratory of Neurogenetics, IRCCS Institute of Neurological Sciences, Bologna, Italy

* Correspondence: daniele.ghezzi@istituto-besta.it

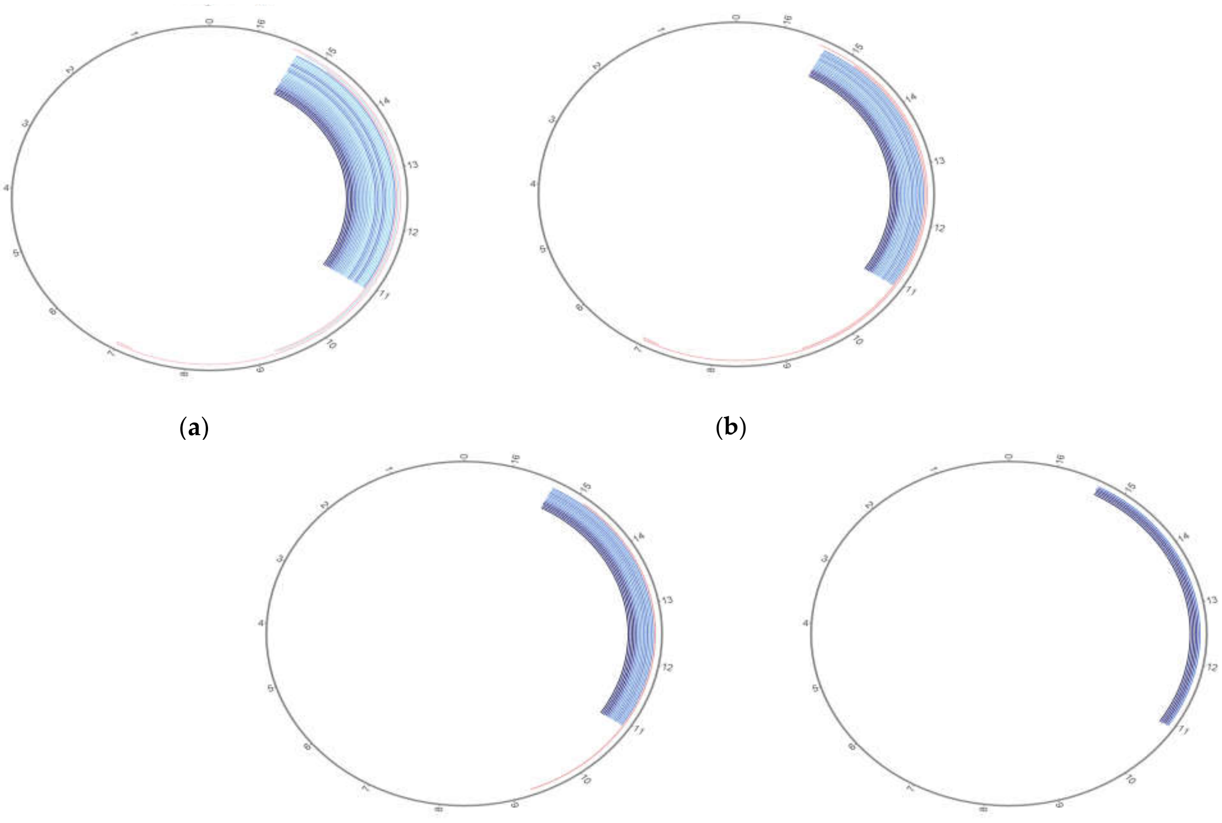

(**a**) (**b**) (**c**) (**d**)

Figure S1. Impact of progressive downsampling on secondary events reduction. Circular plots illustrate the effect of progressively downsampling the reads from high-coverage sample MT7093M, which initially had a mean coverage of 18600x, to achieve uniform coverage at (**a**) 5000x, (**b**) 2000x, (**c**) 1000x, and (**d**) 500x. As coverage decreases, the number of secondary events associated with the primary single large-scale deletion is significantly reduced. Further downsampling can be applied to reach the optimal number of reads required to reliably detect and display only the primary variant, ensuring greater precision.

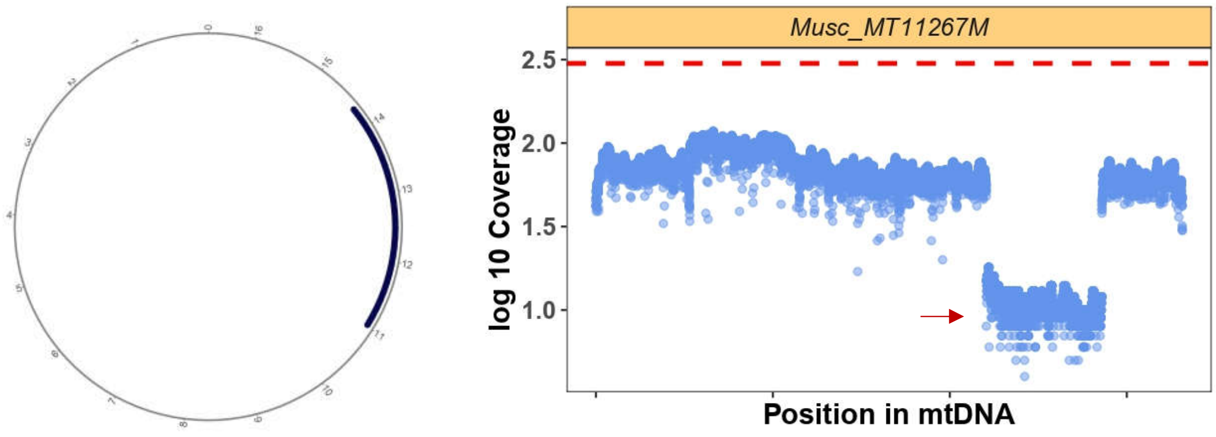

(**a**) (**b**)

Figure S2. Identification of SVs via ONT long-read sequencing.

(a) Circular plot results from MitoSAlt single large-scale deletions analysis in MT11267M sample. MitoSAlt_SE1.1.1.pl algorithm exhibited high precision in pinpointing primary event of 3267 size of bp at major arc, efficiently filtering out secondary events resulting from minor variations in breakpoint positions typically seen in short-read sequencing. In this specific analysis, we adjusted the line thickness parameters in the delplot.R script, which is part of the pipeline, to improve the visualization of the single large deletion in the plot. (**b**) Visualization of large deletions based on the analysis conducted with Mitopore.de, in the section "Note for large deletions". In addition to the notification indicating the presence of a large deletion in sample Musc_MT11267M, the generated plot clearly shows a drop in coverage track, marked by the arrow, at the position corresponding to the expected deletion for the sample (Table 1), further confirming the accuracy and reliability of the Mitopore pipeline in detecting large deletions from long-read sequencing data.

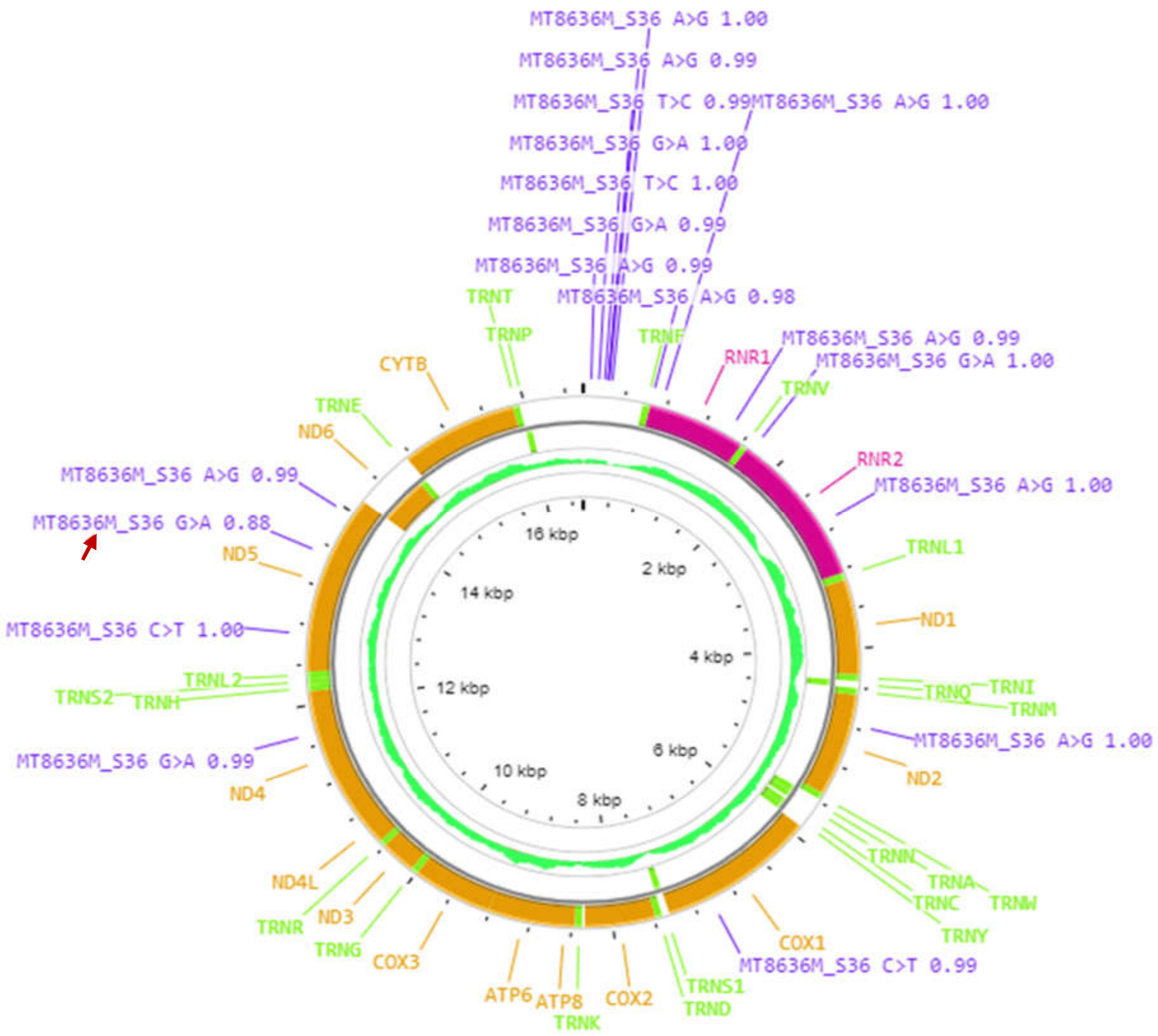

Figure S3. Detailed CGView SNV plot for mtDNA variant analysis in sample MT8636M.
The circular plot displays the distribution of single nucleotide variants (SNVs) across the mitochondrial genome, with annotations indicating relevant genes and genomic regions. Each variant is labeled with its specific nucleotide change and heteroplasmy level. The variant indicated by the arrow (G>A at 0.88 heteroplasmy in the ND5 region) is highlighted as a point of particular interest, potentially associated with pathogenic effects. The interactive, zoomable design allows users to closely examine individual variants, supporting both diagnostic interpretation and broader comparative analysis.

Table S1. Detailed list of mtDNA single nucleotide variants (SNVs) detected in sample MT8636M.

	#CHROM	POS	REF	ALT	FILTER	FORMAT	MT8636M_S36.bam
0	chrM	73	A	G	PASS	GT:AF:DP	1:0.995:1554
1	chrM	143	G	A	PASS	GT:AF:DP	1:0.991:1540
2	chrM	195	T	C	PASS	GT:AF:DP	1:0.997:1580
3	chrM	225	G	A	PASS	GT:AF:DP	1:0.996:1393
4	chrM	226	T	C	PASS	GT:AF:DP	1:0.994:1392
5	chrM	235	A	G	PASS	GT:AF:DP	1:0.994:1573
6	chrM	263	A	G	PASS	GT:AF:DP	1:0.998:1490
7	chrM	648	A	G	PASS	GT:AF:DP	1:0.981:1477
8	chrM	750	A	G	PASS	GT:AF:DP	1:0.996:1789
9	chrM	1438	A	G	PASS	GT:AF:DP	1:0.995:2024
10	chrM	1719	G	A	PASS	GT:AF:DP	1:0.997:2380
11	chrM	2706	A	G	PASS	GT:AF:DP	1:0.997:1565
12	chrM	3106	C	A	PASS	GT:AF:DP	0/1:0.345:58
13	chrM	4769	A	G	PASS	GT:AF:DP	1:0.996:2023
14	chrM	6221	T	C	PASS	GT:AF:DP	1:0.994:1865
15	chrM	6371	C	T	PASS	GT:AF:DP	1:0.996:2112
16	chrM	7028	C	T	PASS	GT:AF:DP	1:0.994:1989
17	chrM	7389	T	C	PASS	GT:AF:DP	1:0.997:1542
18	chrM	8860	A	G	PASS	GT:AF:DP	1:0.997:2076
19	chrM	11719	G	A	PASS	GT:AF:DP	1:0.995:1898
20	chrM	12705	C	T	PASS	GT:AF:DP	1:0.997:2105
21	chrM	13513	G	A	PASS	GT:AF:DP	1/0:0.884:1952
22	chrM	13966	A	G	PASS	GT:AF:DP	1:0.994:1872
23	chrM	14470	T	C	PASS	GT:AF:DP	1:0.993:1358
24	chrM	14766	C	T	PASS	GT:AF:DP	1:0.992:1920
25	chrM	15326	A	G	PASS	GT:AF:DP	1:0.996:2211
26	chrM	16189	T	C	PASS	GT:AF:DP	1:0.981:1597
27	chrM	16192	C	T	PASS	GT:AF:DP	1/0:0.906:1670
28	chrM	16223	C	T	PASS	GT:AF:DP	1:0.987:1812
29	chrM	16278	C	T	PASS	GT:AF:DP	1:0.986:1725
30	chrM	16292	C	T	PASS	GT:AF:DP	1:0.987:1686
31	chrM	16519	T	C	PASS	GT:AF:DP	1:0.996:1557

Each row presents a variant with its chromosomal position (#CHROM, POS), reference (REF) and alternative (ALT) alleles, filter status (FILTER), genotype format (GT), allele frequency (AF), and read depth (DP). The heteroplasmy level is included for each variant, providing insights into variant abundance within the sample, in a format similar to the Mitoverse tool. The variant of interest, G>A at position 13513 with 0.884 heteroplasmy, indicated by the red arrow, is highlighted for its clinical relevance.

Disclaimer/Publisher's Note: The statements, opinions and data contained in all publications are solely those of the individual author(s) and contributor(s) and not of MDPI and/or the editor(s). MDPI and/or the editor(s) disclaim responsibility for any injury to people or property resulting from any ideas, methods, instructions or products referred to in the content.
